# The patients with multiple myeloma were infected with COVID-19 during autologous stem cell transplantation: case report and literature review

**DOI:** 10.1186/s13027-024-00587-2

**Published:** 2024-05-27

**Authors:** Chang Su, Lijun Huang, Liang Liang, Lijia Ou, Guige Lu, Caiqin Wang, Yizi He, Ruolan Zeng, Yajun Li, Hui Zhou, Ling Xiao

**Affiliations:** 1 Department of Lymphoma & Hematology, The Affiliated Cancer Hospital of Xiangya School of Medicine, Central South University/Hunan Cancer Hospital, No. 283 Tongzipo Road, Yuelu District, Changsha, Hunan China; 2 Department of Histology and Embryology, Xiangya School of Medicine, Central South University, No. 172 Tongzipo Road, Yuelu District, Changsha, Hunan China; 3 Graduate Collaborative Training Base of Hunan Cancer Hospital, Hengyang Medical School, University of South China, Hengyang, Hunan China

**Keywords:** Multiple myeloma, COVID-19, ASCT, Immunodeficiency, Hematological diseases

## Abstract

This paper introduces two cases of multiple myeloma, COVID-19 infection during autologous stem cell transplantation, the treatment process, and different results of the two patients, which provides a reference for how to carry out ASCT safely during the COVID-19 normalization stage.

## Introduction

Multiple myeloma (MM) is a malignant tumor of plasma cells that accumulates in bone marrow, leading to renal failure, hypercalcemia, bone destruction, and anemia caused by bone marrow failure. It accounts for 1% of all cancers and 10% of all hematological malignancies [1, 2].

About 588,000 people worldwide have been diagnosed with MM every year. MM usually affects the elderly; the median age at diagnosis is 69 years old [3]. Newly diagnosed MM is usually sensitive to many cytotoxic drugs, and the treatment is mainly autologous hematopoietic stem cell transplantation (ASCT) after induction chemotherapy [4]. After ASCT, the non-recurrent mortality rate was as low as 1% at 12 months, while the recurrence rate was 16%^5^. Median OS with ASCT was significantly better than without transplantation (7.04 vs. 2.32) [6]. Although the treatment scheme containing new drugs has improved OS, ASCT is still an important treatment to prolong OS [5, 7].

Since 2019, the novel coronavirus (SARS-CoV-2) has had a great impact on the world. The mortality rate of elderly patients and/or patients with complications can be as high as 15% [8]. The outcome of COVID-19 in patients with hematological diseases such as leukemia, lymphoma, and myeloma and recipients of autologous (ASCT) or allogeneic hematopoietic stem cell transplantation (allo-SCT) has attracted much attention because of its high humoral and cellular immunosuppression. According to recent research, the total mortality rate related to COVID-19 in patients with hematological diseases is 32 to 40% [9, 10]. Studies have shown that the mortality rate of hospitalized patients infected with MM and COVID-19 is higher than that of non-myeloma patients, and the mortality rate of patients with multiple myeloma in COVID-19 is higher (34%) than that of patients with age and gender-matched non-multiple-myeloma in COVID-19 (23%) [11, 12]. A recent publication in France confirmed that the total mortality rate of all hospitalized COVID-19 patients was 16%, which was significantly lower than the mortality rate (39%) observed among hospitalized patients with multiple myeloma in France [13]. In the post-pandemic era, the management of patients with multiple myeloma and COVID-19 is still based on prevention and antiviral treatment [16]. Although the SARS-COV-2 vaccine can prevent infection, due to the disease state of MM, the patients show diminished response to the SARS-COV-2 vaccine [14, 15]. It was indicated that MM may be more vulnerable to infection, owing to higher rates of mild infections caused by new variants or sub-variants of the virus.

## Case presentations

### Case 1

**Fig. 1:**
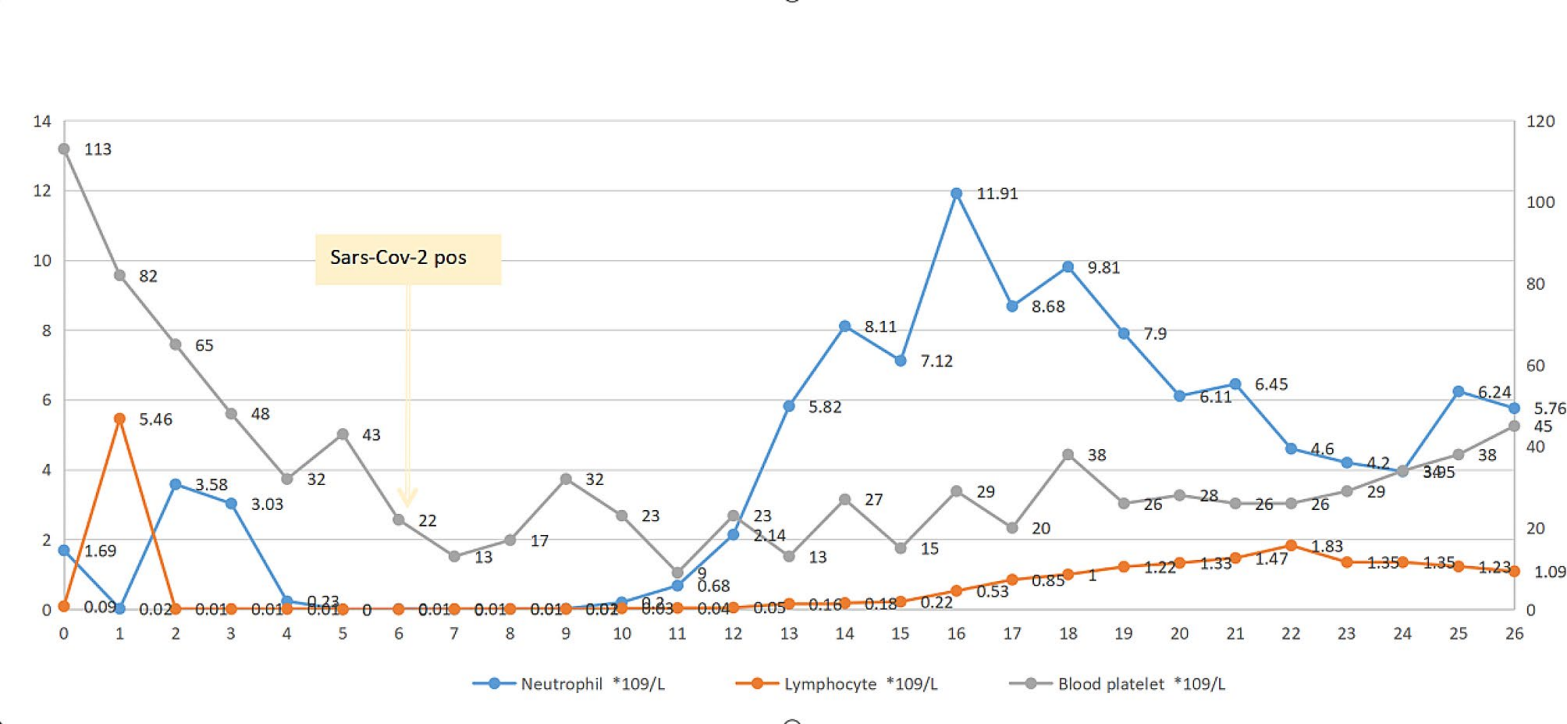
Neutrophil and lymphocyte count and SARS-CoV-2 detection time. The abscissa indicates the days from ASCT

The patient was a 65-year-old male. In September 2022, he was diagnosed with IGg-κ multiple myeloma, DS III stage, and R-ISS III stage. The patient received the first and second cycles of PAD chemotherapy on September 30, 2022, and October 26, 2022, respectively. On November 25, 2022, and January 6, 2023, DVRD chemotherapy was performed, and the efficacy evaluation reached PR. The patient started chemotherapy with 280 mg melphalan on May 16th, 2023, and an autologous stem cell transfusion was performed the next day. On May 23rd, 2023, the patient began to have a fever, and the highest body temperature reached 38℃. The nucleic acid test of throat swab showed that SARS-CoV-2 was positive. Moxifloxacin was combined with sulbactam/cefoperazone to prevent infection, posaconazole was used to prevent fungal infection, and nirmatrelvir /ritonavir tablets were used to prevent viral pneumonia. On May 29th, white blood cells began to rise. On June 1st, the patient’s highest temperature was 38.4℃, the absolute value of neutrophils was 7.12*10^9^/L, and the platelet was 15 * 10^9^/L (Fig. 1). Moxifloxacin was stopped, and linezolid combined with imipenem was used to fight the infection. On the same day, the patient was transferred out of the transplant warehouse and then continued to raise platelets by means of herombopag and platelet transfusion. By June 7, the patient had no fever and chills, and his platelets continued to rise. On June 12, the patient was discharged from the hospital, and the white blood cell (WBC) classification count was basically normal, and his general condition was good. Until October 5, 2023, he continued to maintain treatment with Lenalidomide.

### Case 2

**Fig. 2:**
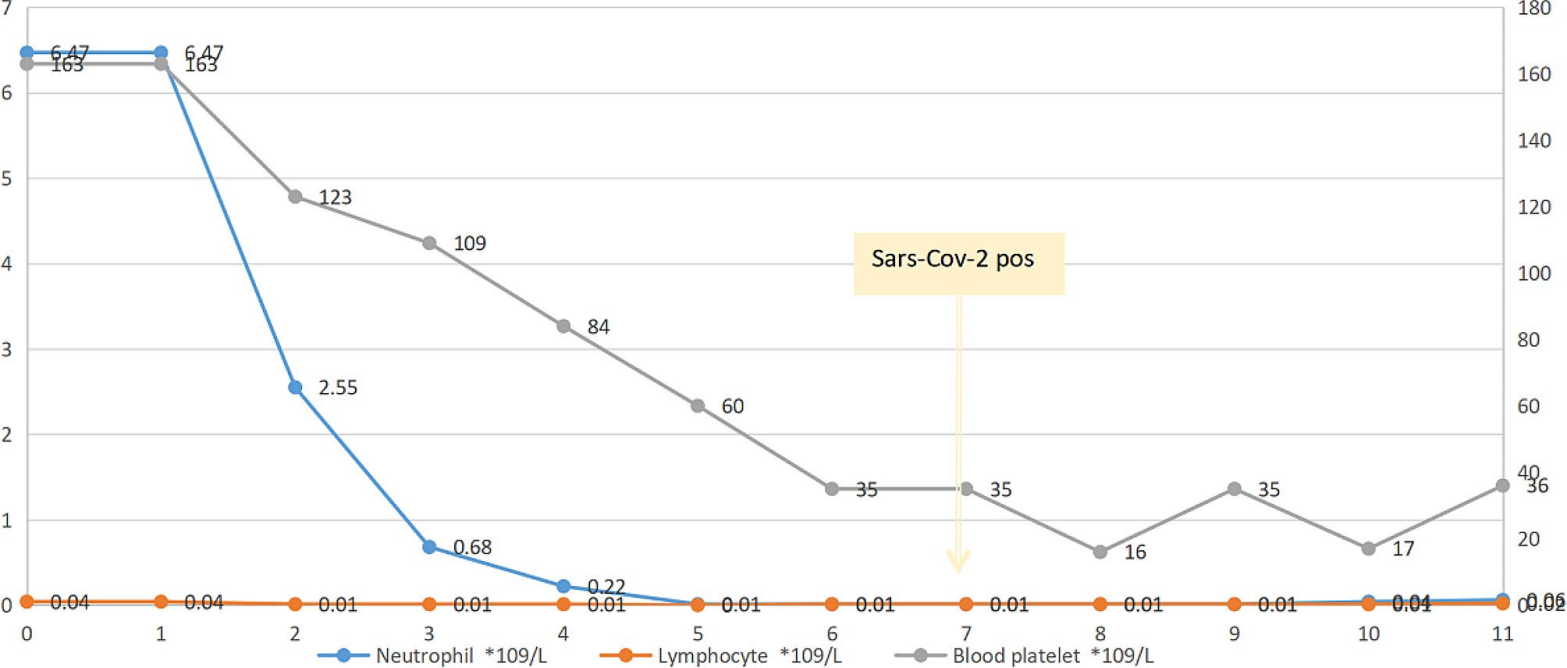
Neutrophil and lymphocyte count and SARS-CoV-2 detection time. The abscissa indicates the days from ASCT

The other patient is a 57-year-old female who was diagnosed with IGg-κ multiple myeloma on January 4, 2023, with DS III stage and R-ISS III stage. From January 4, 2023, to March 27, 2023, four cycles of VRD regimen were performed, and the curative effect reached PR. The patient was admitted to the hospital again in June 2023, during which the SARS-CoV-2 nucleic acid test was positive. After knowing the risk of chemotherapy, he was transferred to a general hospital for antiviral pneumonia treatment, and after the situation improved, he underwent another VRD chemotherapy. On July 19, 2023, after receiving 300 mg of melphalan chemotherapy, autologous stem cells transfusion was performed on July 21. However, the patient developed IV degree bone marrow suppression. Cefoperazone sodium and sulbactam sodium were added to the treatment to prevent infection (Fig. 2). On July 26th, the patient developed a fever, and the highest body temperature reached 38.5℃. The next day, the patient developed symptoms of dyspnea and hypoxemia. Laboratory tests showed that the patient was positive for SARS-CoV-2, cytomegalovirus, influenza A virus, and influenza B virus.Therefore, the patient continues to receive targeted anti-infection treatment. On August 1, the patient had intermittent fever, and his dyspnea did not improve. The patient requested to be transferred to the hospital for treatment and died more than ten days after discharge. The details are unknown.

## Discussion

Due to the disease state of patients with multiple myeloma, COVID-19 vaccine injection can not make patients with multiple myeloma reach the same reaction intensity as healthy people. In the post-epidemic era in COVID-19, people’s awareness of protection declined [14, 15]. Therefore, patients with multiple myeloma are still at high risk of infection.

Previously, among MM patients who received stem cell transplantation, the reported mortality of allo-SCT and ASCT recipients within 30 days after COVID-19 diagnosis was 32% and 33%, respectively [17]. Because of the late immunosuppression of ASCT, it is generally believed that stem cell collection and any transplantation should not be carried out for at least 14 days, preferably 21 days, after the last contact in the case of close contact with people diagnosed with COVID-19. The patients described in this paper obtained COVID-19 in the community and underwent ASCT in the incubation period. Therefore, it may be worthy of attention for doctors to carefully screen whether patients have COVID-19 infection before ASCT.

The treatment strategy of the two patients is similar, but the outcome is quite different. The direct reason is that the second patient’s bone marrow suppression caused by myeloablative therapy before transplantation failed to improve. The patient was infected by multiple pathogens, including cytomegalovirus. Studies have shown that cytomegalovirus infection after ASCT is related to a short OS [18]. The vicious cycle of infection triggered by the sluggish growth of neutrophils and lymphocytes after ASCT, and it may be a significant contributing factor to the ultimate fatality.

Undoubtedly, active anti-SARS-CoV-2 and infection prevention treatment are the keys to patients’ rehabilitation. Infections are a major source of morbidity and mortality among patients with MM [19–21]. There is a case report that an MM female patient was infected with COVID-19 after receiving ASCT and was admitted to the hospital for treatment. After SARS-CoV-2 nucleic acid turned negative, she still died of respiratory failure caused by fungal pneumonia [22]. Immunodeficiency is an inherent problem of MM patients, which may be one of the reasons why the mortality rate of MM and COVID-19-infected inpatients is higher than that of non-myeloma patients [11, 23, 24]. Abnormal humoral immunity makes myeloma patients easy to be complicated with infection [13].In the two cases introduced in this paper, the serum IgG of the first patient before infection was 1961 mg/dl, while that of the second patient was only 669 mg/dL. A previous report pointed out that severe inflammatory reaction to novel coronavirus and severe hypogammaglobulinemia (IgG < 400 mg/dL) was associated with higher mortality [25].

In a word, the possibility of COVID-19 infection must be ruled out during ASCT. During ASCT, the death of patients infected with COVID-19 is caused by many factors, but the main reasons are low immunity and secondary infection. However, how to save the patient in the case of bone marrow transplantation needs further discussion.

## Data Availability

No datasets were generated or analysed during the current study.
